# Cardiovascular Function in Intensive Care Medicine or* Homo Mensura Est*

**DOI:** 10.1155/2016/6301074

**Published:** 2016-04-04

**Authors:** Mitja Lainscak, Zsolt Molnar, Xavier Monnet, Gorazd Voga

**Affiliations:** ^1^Department of Cardiology and Department of Research and Education, General Hospital Celje, Oblakova 5, 3000 Celje, Slovenia; ^2^Faculty of Medicine, University of Ljubljana, Vrazov trg 2, 1000 Ljubljana, Slovenia; ^3^Department of Anesthesiology and Intensive Therapy, Faculty of Medicine, University of Szeged, 6 Semmelweis Street, Szeged 6725, Hungary; ^4^AP-HP, Hôpitaux Universitaires Paris-Sud, Hôpital de Bicêtre, Service de Réanimation Médicale, 94270 Le Kremlin-Bicêtre, France; ^5^Université Paris-Sud, Faculté de Médecine Paris-Sud, Inserm UMR U-999, 94270 Le Kremlin-Bicêtre, France; ^6^Medical ICU, General Hospital Celje, Oblakova 5, 3000 Celje, Slovenia

Global burden of disease, in terms of both mortality and morbidity, is increasing [1, 2]. Ageing of population and better management of acute conditions are significant contributors, yet there is much more to be done. Also, there are numerous situations in clinical practice where we are left with limited evidence and various degrees of uncertainty regarding how to deliver the best medical practice to our patients. This in particular applies for emergency and intensive care where decisions need to be taken within minutes if not seconds; it is therefore not surprising that practicing physician may take suboptimal ways to handle the clinical challenges [3]. Heart failure serves as a good example and in fact this is only cardiovascular condition with increasing prevalence [4]. By adding various comorbidities to the main disease (e.g., anaemia, chronic obstructive pulmonary disease, chronic kidney disease, and cancer, all potentially with body wasting), you end up with an individual prone to deterioration of one or more components of its health status [5–9]. An additional challenge in acute deterioration of chronic disease is iatrogenic due to complex pharmacological therapy; in its essence, very best incentive for a stable patient can get really cumbersome for patient and a clinician once faced with failing organ functions, a common scenario in emergency and intensive medicine. With changes in pharmacokinetics, pharmacodynamics effects are unpredictable, as may be the drug-drug and in particular drug-disease or drug-disease-drug interactions [10–12]. Adding the immediate-acting drugs we usually use in this setting, we are indeed exploring the limited-evidence land and outcomes are less predictable. This, in fact, reminds us once again that we need to consider every patient as an individual with peculiarities and specific response to disease/acute condition and management we employ. Herein, we need to get back to the basics or to the fact that* Homo mensura est* (*Homo mensura est* stands for man is the measure said by Protagoras (Greek: Π*ρωταγ*ό*ρας*, c. 490–c. 420 BC)) or, in other words, that medicine is an art [13]. Yet, this coin has two sides: one is the patient whilst the other one is the practicing physician. And both of them are humans, with all pros and cons. Once in hospital due to acute condition or with critical illness, a comprehensive evaluation of cardiovascular system is crucial for reliable assessment of disease severity and management steps. Here, the other side of the coin, namely, the practicing physician, is taking Centre stage. Again,* Homo mensura est* is more true than ever. With all difficulties to assess the cardiovascular function and to take decisions in best of patient interest, one needs to rely on parameters one can reliably assess and interpret. This largely depends on one's training, experience, and confidence with particular monitoring tools or biomarkers [14–16]. Unfortunately, clinical practice tells us, despite all efforts by the clinical community [17, 18], that we do not meet the standards of good clinical practice [19]. Counterintuitively, the development of less invasive methods for assessment of haemodynamic parameters (that all have limitations in the critically ill) did not change patterns of invasive haemodynamic monitoring. Despite availability, echocardiography remains critically underused for these purposes. The lesson learned from the FENICE study [19] should be considered as an important signal to fine-tune our preclinical and clinical training to optimize patient assessment and management.

In acute conditions, we are indeed left with limited-evidence-based medicine. But are there ways to overcome this? Primarily, we should not have prejudices how to handle our patients. The story of beta-blockers in heart failure might serve as a useful example. Initially these drugs were contraindicated, but in current heart failure guidelines, the largest wealth of evidence for prognostic benefit lies within them. Indeed, we are trying to break some long-lasting taboos of misconception through use of these pharmacological agents in obstructive pulmonary disease and during acute deterioration [20–23]. A similar frontier is sepsis, for example, [24]. In sepsis, a closer cooperation between scientific communities, clinicians, and regulatory agencies is required in order to meet future challenges. Some other communities have already paved the way and it is our mission to follow in their footsteps [25]. This issue tried to address some of these aspects. Indeed, we feel that education aiming to optimize patient assessment and to understand the pathophysiological rationale of our actions is crucial in our striving to improve patient outcome. In clinical practice, various options for haemodynamic assessment should be available. Accurate measurement gives us reliable findings that are the basis for timely diagnosis and therapeutic decisions, tailored to individual patient. We would therefore like to promote this special issue and the two review articles by J. Benes et al. and H. A. Gaspar and S. S. Morhy in particular; furthermore, we would once again like to underline the Protagoras quote* Homo mensura est*. Although generally applicable, the significance in emergency and intensive care medicine may be particularly relevant.

*Mitja Lainscak* *Zsolt Molnar* *Xavier Monnet* *Gorazd Voga*
